# Four to seven random casual urine specimens are sufficient to estimate 24-h urinary sodium/potassium ratio in individuals with high blood pressure

**DOI:** 10.1038/jhh.2015.84

**Published:** 2015-08-27

**Authors:** T Iwahori, H Ueshima, S Torii, Y Saito, A Fujiyoshi, T Ohkubo, K Miura

**Affiliations:** 1 Research and Development Department, OMRON HEALTHCARE Co., Ltd, Muko, Japan; 2 Department of Public Health, Shiga University of Medical Science, Otsu, Japan; 3 Center for Epidemiologic Research in Asia, Shiga University of Medical Science, Otsu, Japan; 4 Department of Hygiene and Public Health, Teikyo University School of Medicine, Tokyo, Japan

## Abstract

This study was done to clarify the optimal number and type of casual urine specimens required to estimate urinary sodium/potassium (Na/K) ratio in individuals with high blood pressure. A total of 74 individuals with high blood pressure, 43 treated and 31 untreated, were recruited from the Japanese general population. Urinary sodium, potassium and Na/K ratio were measured in both casual urine samples and 7-day 24-h urine samples and then analyzed by correlation and Bland–Altman analyses. Mean Na/K ratio from random casual urine samples on four or more days strongly correlated with the Na/K ratio of 7-day 24-h urine (*r*=0.80–0.87), which was similar to the correlation between 1 and 2-day 24-h urine and 7-day 24-h urine (*r*=0.75–0.89). The agreement quality for Na/K ratio of seven random casual urine for estimating the Na/K ratio of 7-day 24-h urine was good (bias: −0.26, limits of agreements: −1.53–1.01), and it was similar to that of 2-day 24-h urine for estimating 7-day 24-h values (bias: 0.07, limits of agreement: −1.03 to 1.18). Stratified analyses comparing individuals using antihypertensive medication and individuals not using antihypertensive medication showed similar results. Correlations of the means of casual urine sodium or potassium concentrations with 7-day 24-h sodium or potassium excretions were relatively weaker than those for Na/K ratio. The mean Na/K ratio of 4–7 random casual urine specimens on different days provides a good substitute for 1–2-day 24-h urinary Na/K ratio for individuals with high blood pressure.

## Introduction

Worldwide, reducing salt intake and increasing potassium intake are important measures to reduce blood pressure.^1^ Many guidelines for the prevention and treatment of hypertension recommend reduction of daily salt intake; for example, WHO guideline says <5 g per day.^2, 3, 4, 5, 6^ In spite of the rigorous campaigning and recommendations for salt restriction, however, a fairly large gap continues to exist between the recommended target levels and actual salt intake among populations.^7, 8, 9^

Previous findings show that awareness of salt restriction is not sufficient for actual salt reduction in individuals.^10, 11^ Effective monitoring of adherence to the recommended dietary salt and potassium intake in hypertensive patients and general populations requires development of a convenient, inexpensive and appropriate monitoring system that will make each individual aware of his or her salt intake level and support dietary improvement habits.

The gold standard for estimating an individual's daily salt intake and potassium intake is 24-h urine collection.^12, 13, 14, 15, 16^ To estimate the true long-term sodium intake, 24-h urine collection expanded for several days provide more reliable estimate of a person's salt consumption levels rather than single 24-h urine collection, as the day-to-day variation in sodium intake and its urine excretion are relatively high.^16, 17^ In addition, the sodium/potassium (Na/K) ratio in 24-h urine has been reported to be related to blood pressure in epidemiologic studies.^18, 19, 20, 21, 22^ Recent data from the observational studies reviewed provide additional support for the Na/K ratio as a superior metric to either sodium or potassium alone in the evaluation of blood pressure outcomes and incident hypertension.^22, 23^ However, repeated 24-h urine collections are neither easy nor practical for patients at clinics or at home.

In our previous study, we have found that the mean Na/K ratio of six random daytime casual urine samples showed a strong correlation with and good agreement with the mean 7-day 24-h urinary Na/K ratio in healthy Japanese participants, mainly in normotensive individuals.^24^ However, the accuracy of repeated measurements of casual urine for the estimation of Na/K ratio has not been investigated in individuals with high blood pressure including treated hypertensives.

This study aimed to clarify the optimal number and type of casual (spot) urine specimens required for suitable estimation of individual daily Na/K ratio on different days in high blood pressure individuals using 7-day 24-h urine collection as the gold standard.

## Materials and methods

### Participants and measurements

A total of 74 men and women with stage 1 hypertension or high normal blood pressure (systolic/diastolic blood pressure over 130/80 mmHg), 43 treated and 31 untreated among ages 40–69 years, were recruited from among high blood pressure volunteers living in Kyoto, Japan and surrounding areas. Menstruating women; individuals with secondary hypertension, diabetes or chronic kidney disease; and individuals with a history of diabetes, cardiovascular disease, cerebrovascular disease or chronic kidney disease were excluded. Participants were instructed to collect all urine samples and to measure urine volume with a standardized measuring cup at each voiding for a minimum of 7 consecutive days, unless urine collection was unsuccessful or contaminated by feces. They were instructed that 1-day urine collection began when they woke up and ended at wake-up the following morning to prevent miss collection of casual urine. At the calculation of 24-h urinary excretion of a day, the first void of the day was excluded and the first void of the next day was included. If participants declared that they failed to complete urine collection, we asked them to retry urine collection. Urine samples were first collected in a standardized measuring cup for urine volume measurement, and then a part of urine was transferred into an aliquot tube (10 ml) at each voiding for the measurement of sodium and potassium concentrations. Each void was collected in a separate aliquot tube. The number of casual urine samples obtained during the 7 days was dependent on voiding frequency of each individual. All casual urine samples were sent to the central laboratory at room temperature for measurement.

Participants were asked to record the volume (ml) of each sample and the time of collection. Participants were required to take blood samples, measure their height, weight, blood pressure. The blood samples were obtained from the participants by trained staff after the blood pressure measurement, and participants were requested to start fasting minimum 8 h prior to taking blood samples. Baseline blood pressure was measured twice by trained observers using blood pressure monitor (HEM-7081IT, OMRON Healthcare, Kyoto, Japan) on the right arm of seated participants after at least 5 min of rest. The mean values of the two blood pressure measurements were used for analysis. Written informed consent was obtained from all participants. The ethics committee of the Shiga University of Medical Science and Omron Healthcare Company approved the study protocol.

At the central laboratory, sodium and potassium concentrations (mmol l^−1^) in all urine samples were analyzed by ion-selective electrodes at a third party's lab (BML Inc., Tokyo, Japan). The 24-h urinary sodium and potassium excretion (mmol per 24-h) was calculated from the sum of urinary excretion at each voiding over 1 day. The Na/K ratio of each casual urine sample was calculated using the sodium and potassium concentrations of each casual urine sample, and the 24-h urinary Na/K ratio was calculated using 24-h urinary excretion of sodium and potassium for that day. The timing of casual urine was defined as follows: (1) first morning urine: first voiding after rising, (2) second morning urine: second voiding after rising, (3) random daytime casual urine: specimen randomly selected from urine voided between 09 and 17 h, (4) random casual urine: urine specimen randomly selected among all urine specimens on a certain day, (5) urine before bedtime: urine voided approximately 8 h before the next first morning voiding. Only one sample per day was randomly selected by the computer program as the random casual urine specimen for that day. The mean value of 24- h urinary Na/K ratios for 7 days for each participant was used as the gold standard for individual urinary Na/K ratio. The casual urine specimens were chosen from same 7 consecutive days as 7-day urine collection was done. These casual urine specimens were also chosen from the first day, the first 2 days, the first 3 days and so on, until 7 consecutive days to clarify the optimal number of required casual urine specimens for suitable estimation of the gold standard. Body mass index was calculated as weight divided by height squared (kg m^−2^) and estimated glomerular filteration rate was calculated as 194 × serum creatinine^−1.094^ × age^−0.287^ × 0.739 (if female) (ml  min^−1^ per 1.73 m^2^).^25^

### Statistical analysis

Mean Na/K ratio, sodium concentration, and potassium concentration in casual urine samples were calculated for the first day, the first 2 days, the first 3 days, and so on, for 7 successive days after the beginning of urine collection for each individual. The same calculations were performed for the first morning urine, the second morning urine, random daytime casual urine, the urine before bedtime and random casual urine, respectively.

Pearson's correlation coefficients for the Na/K ratio were calculated to examine the correlation between the specific values for casual urine and the corresponding mean values for 24-h urine samples over a 7-day period as the gold standard. For example, correlation coefficients for the first morning urine Na/K ratio (value of the first 1 day, mean of the first 2 days, similarly those of successive days and mean of all 7 days) were made with the mean 7-day 24-h urine Na/K ratio. The calculations were performed for the daily first morning urine, second morning urine, random daytime casual urine, urine before bedtime and random casual urine samples. Correlations were also calculated between the sodium and potassium concentration of casual urine and the mean 24-h sodium and potassium excretion over 7 days.

Agreement between the casual urine Na/K ratio and 7-day 24-h urine Na/K ratio was examined using the method proposed by Bland and Altman.^26^ The ‘agreement', estimated by bias (mean difference) and the upper and lower limits of agreement (bias±1.96 × s.d. of difference) between the casual urine method and 7-day 24-h urine method, was also compared with that between some repeated 24-h urine Na/K ratio and 7-day 24-h urine one. Statistical analyses were conducted using the general linear models procedure and the logistic regression procedure in SAS 9.3 for these evaluations.

## Results

The characteristics and urinary findings of the study participants are shown in Table 1. The mean age of participants was 58.4 years. Thirty-five participants (47.3%) were women. The mean voiding frequency was 7.57 times per day and the mode was seven times per day. Of the random casual urine samples, 14% were first morning urine samples, 15% were second morning urine samples, 15% were urine before bedtime samples and 55% were other samples. Mean 24-h urinary volume for 7 days was 1799 ml. Mean 24-h sodium excretion over 7 days was 195.3 mmol per 24 h, whereas mean 24-h potassium excretion was 59.6 mmol per 24 h. Casual urine sodium concentrations and casual urine potassium concentrations were lowest in the first morning urine. The mean Na/K ratio of 24-h urine was 3.43. Casual urine Na/K ratio was lowest in the second morning urine sample.

Of the individuals on antihypertensive medications, 14 participants (33%) were taking calcium channel blockers, 9 participants (21%) were taking angiotensin 2 receptor blockers, 15 participants (35%) were taking both calcium channel blockers and angiotensin 2 receptor blockers and 5 participants (12%) were taking other drugs.

Correlation coefficients of the casual urine Na/K ratio with 7-day 24-h Na/K ratio, sodium excretion and potassium excretion in the 74 individuals are shown in Table 2. The correlation between casual urine Na/K ratio and 7-day 24-h Na/K ratio generally became stronger as the number of days increased. In terms of the time of casual urine, correlation coefficients were generally the highest for random casual urine samples as compared with the first morning, second morning, before bedtime and random daytime casual urine samples. Values were 0.80–0.87 for 4 or more days of random casual urine samples, 0.61–0.63 for first morning samples, 0.67–0.72 for second morning samples, 0.66–0.68 for before bedtime samples and 0.76–0.85 for random daytime casual urine samples (*P*<0.001 for all coefficients). The Na/K ratio of the random daytime casual urine had the second strongest correlation with 24-h Na/K ratio after random casual urine (Table 2). Stratified analyses comparing individuals taking antihypertensive medication and individuals not taking antihypertensive medication showed similar results with respect to the correlation coefficients for the mean of 4 or more days of the casual urine Na/K ratio and 7-day 24-h Na/K ratio, except for the first morning urine and urine before bedtime samples (Table 2).

Casual urine sodium concentration was moderately correlated with 7-day 24-h sodium excretion, although the correlation was weaker than casual urine Na/K ratio with the 7-day 24-h Na/K ratio (Supplementary Appendix Table 1). The correlation coefficient between casual urine sodium concentration and 7-day 24-h urine sodium excretion reached 0.56 for the mean 5-day value of the first morning urine and 0.52 for the mean 6-day random casual urine value (Supplementary Appendix Table 1). The correlation coefficients between casual urine potassium concentration and 24-h urinary potassium excretion reached 0.39 for the mean 5-day value of random daytime casual urine and 0.54 for the mean 7-day random casual urine value (Supplementary Appendix Table 2).

**Figure 1:**
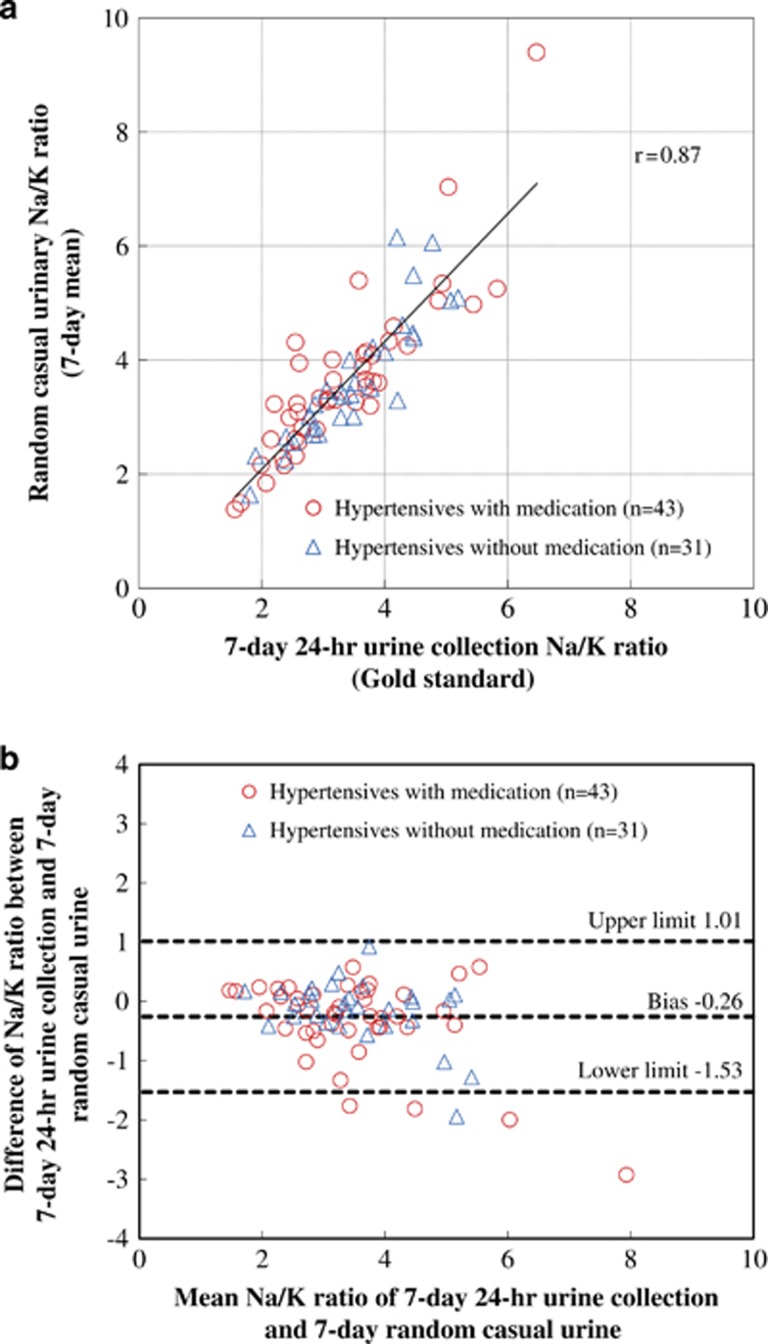
Pearson's correlation coefficient between 7-day random casual urine Na/K ratio and 7-day 24-h urine Na/K ratio for the 74 individuals (**a**). The bias between the 7-day casual urine Na/K ratio and 7-day 24-h Na/K ratio by Bland–Altman method was −0.26, with the limits of differences lying between −1.53 and 1.01 (**b**). The quality of agreement demonstrated by the Bland–Altman method was similar to that in Figure 2, which shows Pearson's correlation coefficient and the bias with the limits of differences, between the 2-day 24-h urine Na/K ratio and 7-day 24-h urine Na/K ratio.

Because the correlation coefficient values for Na/K ratio, Na and K concentrations of casual urine with Na/K ratio, Na and K excretions of 24-h urine collection were highest for the first of these three correlations compared with corresponding *r*-values for casual and 24-h urinary Na or K, assessment of agreement by the Bland–Altman method was performed for the Na/K ratio, especially for the casual urine with the strongest correlation. The correlation coefficient between the Na/K ratios of the random casual urine and 7-day 24-h urine reached 0.80 for the mean 4-day value and 0.87 for the mean 7-day value (Figure 1a, Table 2). The bias for the 4-day casual urine method and 7-day 24-h method was −0.21 and limits of agreements were −1.76 to 1.33. The bias for the 7-day casual urine method and 7-day 24-h method was −0.26 and limits of agreements were between −1.53 and 1.01 (Figure 1b). The comparison of individuals taking antihypertensive medication and individuals not taking antihypertensive medication showed similar results (Figure 1b).

**Figure 2:**
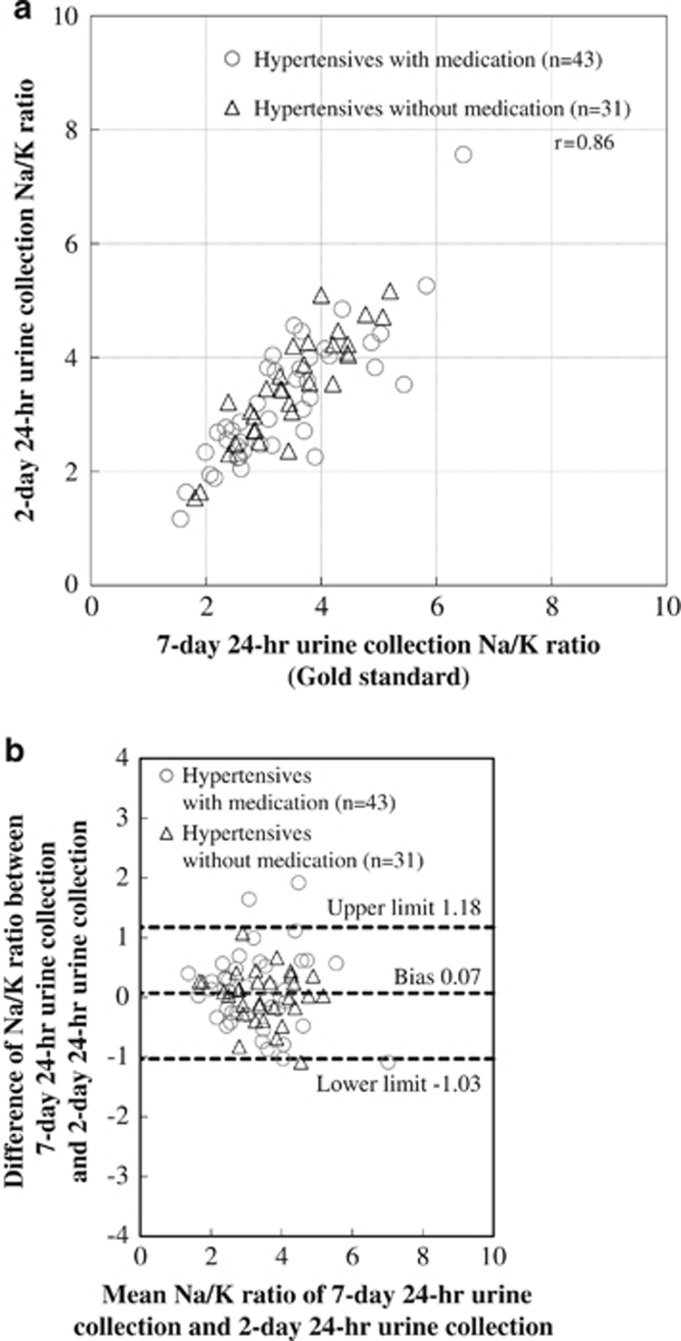
Pearson's correlation coefficient between the 2-day 24-h Na/K ratio and 7-day 24-h Na/K ratio was 0.86 for the 74 individuals (**a**). The bias between the 2-day 24-h urine Na/K ratio and 7-day 24-h urine Na/K ratio by Bland–Altman method was 0.07, with the limits of differences lying between −1.03 and 1.18 (**b**).

The bias and limits of agreement were also compared by the Bland–Altman method between 1 or more days of mean of 24-h Na/K ratios (conventional method) and the 7-day 24-h Na/K ratio (gold standard in this study) as a reference to understand the accuracy of our suggested method. The ‘agreement' of the Na/K ratio of 4-day random casual urine was better than that of the 1-day 24-h urine Na/K ratio (bias: 0.10; limits of agreement: −1.47 to 1.67), whereas the correlation coefficient with the gold standard was 0.75. On the other hand, the ‘agreement' of the Na/K ratio of 7-day random casual urine was almost similar to that of the 2-day 24-h urine Na/K ratio (bias: 0.07; limits of agreement: −1.03 to 1.18), whereas the correlation coefficient of the 2-day 24-h urine Na/K ratio with the gold standard was 0.86 (Figure 2b, Supplementary Appendix Table 3).

## Discussion

We found that in individuals with high blood pressure, assessment of 4–7 random samples of casual urine on different days provides a good estimate of an individual's daily urinary Na/ K ratio, which was similar to that determined by 1–2-day 24-h urine collections. Pearson's correlation coefficient of the Na/K ratios determined by the two types of collection methods (4–7 random samples of casual urine and 1–2-day 24-h urine collections) with 7-day 24-h urinary Na/K ratio (gold standard) were similar (0.80–0.87 and 0.75–0.86). The ‘agreement' defined by the Bland–Altman method was also assessed by the two types of collection methods (4–7 random samples of casual urine and 1–2-day 24-h urine collections) with 7-day 24-h urinary Na/K ratio (gold standard), and both showed similarly good agreement quality. The limits of agreements for Na/K ratio of 4–7 random casual urine samples for estimating Na/K ratio of 7-day 24-h samples was >−1 to 1, where the mean Na/K ratio was 3.43. However, the limits of agreements for Na/K ratio of 1–2-day 24-h urine samples for estimating Na/K ratio of 7-day 24-h samples also showed greater width than –1 to 1. Therefore, our method using Na/K ratio of 4–7 random casual urine samples would be useful for estimating individual value, similarly to 1–2-day 24-h urine collection. The voiding frequency was nearly seven times per day and this might explain the required number of spot urine to estimate the 7-day 24-h urinary Na/K ratio (gold standard) with high correlation and good agreement quality.

Considering our previous report in healthy individuals, this method would be applicable regardless of whether individuals are healthy or have high blood pressure.^24^ The proportion of blood pressure drug types in the individuals taking antihypertensive medication in the present study was similar to that observed in a Japanese general clinic.^11^ Therefore, these findings suggest that this method for assessing salt and potassium intake is acceptable in outpatients. There might be neurohormonal factors or circadian rhythm or physiologic regulation affecting urinary excretion patterns in the Na/K ratio (high in first morning samples and before bedtime samples, and low in daytime samples) for both healthy individuals and individuals with high blood pressure, as our previous study and present study showed similar results.^24^

Moreover, this method is also applicable to individuals with high blood pressure regardless of antihypertensive medication being taken when using random daytime casual samples and random casual urine samples for estimation. Therefore, better estimation for this method requires casual urine collection in diverse time slots, especially avoiding casual urine collection concentrated on first morning and before bedtime.

The study findings suggest this method's applicability as a convenient self-monitoring tool to support individuals in their efforts to reduce salt intake and increase potassium intake. It is well known from the INTERSALT and other studies that the Na/K ratio in 24-h urine is related to blood pressure.^18, 19, 20, 21^ Na/K ratio is also known as a superior metric to either sodium or potassium alone in the evaluation of blood pressure outcomes and incident hypertension.^22, 23^ In comparison with conventional urine collection methods or methods of estimating daily salt intake, use of a random casual urinary Na/K ratio provides higher accuracy for individual estimation.^27, 28^ Nowadays, handy devices for casual urinary Na/K ratio measurement are available that provide prompt feedback with far lower patient burden compared with conventional methods that require either urine collection or urinary creatinine measurements.^27, 28, 29^ Therefore, it is reasonable and practical for patients to use their Na/K ratio to understand their own value and try to reduce salt intake and increase potassium intake through self-monitoring experiences identifying and avoiding high-sodium and low-potassium food source in the daily life for blood pressure control even at home or outside medical office.

A limitation of this study is a relatively small sample size that did not include the complete variety of blood pressure drugs that patients may use. Instead of >60 different drugs in at least 11 separate categories of blood pressure drugs, we primarily included patients taking calcium channel blockers, angiotensin 2 receptor blockers or a combination of a calcium channel blocker and angiotensin 2 receptor blocker.^30^ Hence, it is not known whether these findings are also applicable to individuals taking blood pressure drugs that are not predominantly used in the Japanese general population or in Japanese general clinics, such as diuretics, beta-blockers, angiotensin converting enzyme inhibitors, alpha-blockers, central agonists or combinations of different drugs.

Salt reduction and potassium increase are important worldwide measures to reduce blood pressure, although prior finding shows that awareness of salt restriction is not sufficient for actual salt reduction in individuals.^10, 11^ Conventional tools and methods are not convenient and practical enough for identifying individual values of salt reduction and potassium increase in outside of and even inside of the medical office. The present study findings, using 4–7 random sample measurements of casual urine on different days for estimation of the daily dietary Na/K ratio, is a good substitute for 1–2-day 24-h urine collection not only in healthy individuals but also in high blood pressure individuals regardless of taking blood pressure drugs. By virtue of its ease, convenience and prompt feedback to individuals, this method is also expected to increase the awareness of individual values of salt reduction and potassium increase and guide individuals to proper dietary management through behavior modification, thus controlling hypertension and preventing many of the serious illness associated with this condition. This method may also help clinicians identify proper responses to blood pressure drugs for their patients and help them guide patients to reduce their blood pressure by providing information on dietary management of salt and potassium intake.

The WHO guidelines recommend free-living individuals to satisfy dietary Na/K ratio of <1, although there is no clear recommended cutoff value for urinary Na/K ratio.^6, 31^ Stamler J *et al.*^18^ recommended urinary Na/K ratio 1.0 as a target level from the findings of the INTERSALT. Therefore, we recommend urinary Na/K ratio <1.0 to keep blood pressure lower. Further investigations would be needed to determine optimal cutoff point of urinary Na/K ratio.


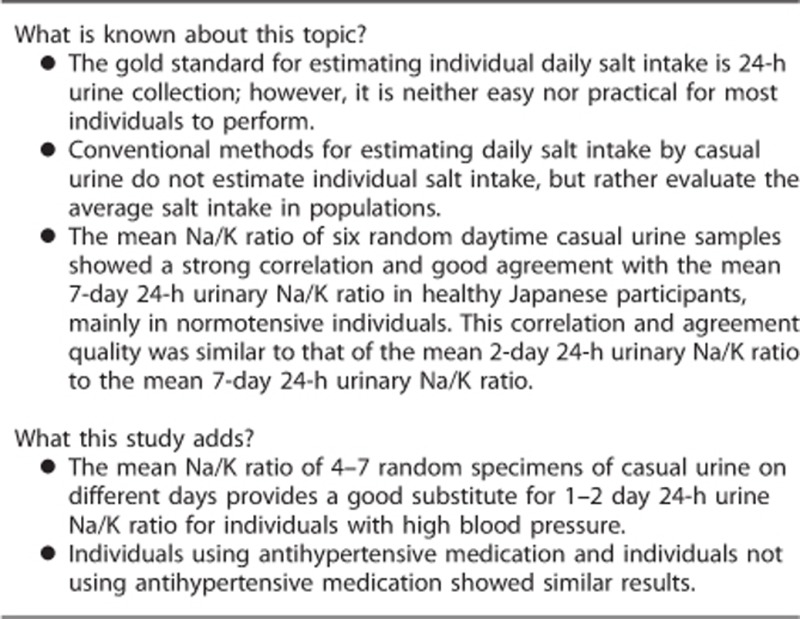


## Figures and Tables

**Table 1:** Characteristics and urinary findings of study participants

*Variables*	*With antihypertensive medication (*n=*43)*	*Without antihypertensive medication (*n=*31)*	*Overall (*n=*74)*
	*Mean*±*s.d.*	*Mean*±*s.d.*	*Mean*±*s.d.*
Age	61.2±7.6	54.3±8.5	58.4±8.7
Height (cm)	161.2±7.2	163.7 ±7.5	162.2±7.4
Weight (kg)	63.5±11.3	64.8±13.1	64.2±12.0
Body mass index (kg m^−2^)	24.3±3.4	24.0±3.7	24.2±3.5
Systolic blood pressure (mmHg)	138.1±13.2**	142.1±8.6	139.8±11.6
Diastolic blood pressure (mmHg)	84.1±7.8	87.6±7.4	85.6±7.8
Serum LDL cholesterol (mg dl^−1^)	129.8±34.3	140.6±42.0	134.3±37.8
Serum triglycerides (mg dl^−1^)	100.7±43.9**	117.3±132.1	107.6±91.3
Serum HDL cholesterol (mg dl^−1^)	66.6±17.0	61.6±16.7	64.5±16.9
Blood glucose (mg dl^−1^)	94.5±12.0*	88.9±9.0	92.2±11.1
HbA1c (%)	5.6±0.4*	5.6±0.6	5.6±0.5
Estimated GFR (ml min^−1^ per 1.73 m^2^)	85.9±14.9*	82.7±10.4	84.6±13.2
Serum uric acid (mg dl^−1^)	5.3±1.3	5.1±1.1	5.2±1.2
			
	n *(%)*	n *(%)*	n *(%)*
*Women (overall)*	*19 (44.2)*	*16 (51.6)*	*35 (47.3)*
24-h urine volume (ml)	1746.8±582.4	1870.8±610.9	1798.8±597.1
24-h Na excretion (mmol per 24 h)	187.7±72.8	205.9±75.2	195.3±74.3
24-h K excretion (mmol per 24 h)	59.3±18.7	59.9±18.3	59.6±18.5
Urine voiding frequency (no. of voids per day)	7.9±2.2**	7.2±1.7	7.6±2.1

*Na concentration (mmol l^−1^)*			
24-h urine	112.8±41.0	115.5±42.2	113.9±41.5
1st morning urine	104.3±46.7	114.1±46.8	108.4±47.0
2nd morning urine	116.0±50.1	123.2±47.3	119.0±49.0
Random daytime casual urine	121.0±52.0	120.1±52.2	120.6±52.0
Urine before bedtime	117.4±60.7	133.1±60.1	124.0±60.9
Random casual urine	117.2±53.0	119.2±55.5	118.1±54.0

*K concentration (mmol l^−1^)*			
24-h urine	36.3±13.1	34.4±12.1	35.5±12.7
1st morning urine	33.4±18.7**	28.2±14.2	31.2±17.1
2nd morning urine	47.0±23.7	47.1±24.3	47.0±24.0
Random daytime casual urine	44.7±24.3**	42.3±21.1	43.7±23.1
Urine before bedtime	40.3±26.5**	37.3±19.9	39.1±24.0
Random casual urine	41.0±23.8**	37.5±21.2	39.5±22.8

*Na/K ratio*			
24-h urine	3.35±1.37**	3.53±1.11	3.43±1.27
1st morning urine	3.92±2.54**	4.59±2.15	4.20±2.40
2nd morning urine	3.21±2.54**	3.10±1.48	3.16±2.16
Random daytime casual urine	3.37±2.24**	3.24±1.51	3.32±1.97
Urine before bedtime	3.79±2.37**	4.17±2.03	3.95±2.24
Random casual urine	3.65±2.37**	3.79±1.98	3.71±2.21

Abbreviations: GFR, glomerular filtration rate; HbA1c, hemoglobin A1c; HDL, high-density lipoprotein; K, potassium; LDL, low-density lipoprotein; Na, sodium.

Urinary findings are the means of the 7 days.

*P<0.05, **P<0.01 vs individuals not taking antihypertensive medication.

**Table 2:** Correlation coefficients of casual urine Na/K ratio with 7-day 24-h Na/K ratio, Na excretion and K excretion in 74 hypertensive participants

*Time of casual urine*	*Number of days to calculate mean*	*Correlation coefficients*								
		*24-h Na/K ratio*^a^			*24-h Na excretion*^a^ *(mmol per 24 h)*			*24-h K excretion*^a^ *(mmol per 24 h)*		
		*With medication (*n=*43)*	*Without medication (*n=*31)*	*Overall (*n=*74)*	*With medication (*n=*43)*	*Without medication (*n=*31)*	*Overall (*n=*74)*	*With medication (*n=*43)*	*Without medication (*n=*31)*	*Overall (*n=*74)*
*First morning urine*										
	1 day	0.33	0.74	0.46	0.23	0.36	0.29	−0.10	−0.24	−0.14
	2 days	0.36	0.73	0.48	0.26	0.40	0.32	−0.12	−0.22	−0.15
	3 days	0.49	0.79	0.59	0.37	0.38	0.38	−0.15	−0.29	−0.19
	4 days	0.54	0.77	0.61	0.41	0.35	0.39	−0.16	−0.29	−0.21
	5 days	0.56	0.79	0.63	0.36	0.39	0.38	−0.22	−0.25	−0.22
	6 days	0.53	0.79	0.62	0.36	0.37	0.38	−0.18	−0.27	−0.21
	7 days	0.51	0.81	0.61	0.36	0.36	0.37	−0.18	−0.31	−0.22
										
*Second morning urine*										
	1 day	0.45	0.45	0.45	0.52	0.24	0.41	0.03	−0.14	−0.02
	2 days	0.38	0.57	0.41	0.31	0.29	0.27	−0.10	−0.17	−0.11
	3 days	0.57	0.60	0.56	0.41	0.30	0.34	−0.19	−0.18	−0.18
	4 days	0.60	0.68	0.60	0.45	0.37	0.39	−0.20	−0.17	−0.19
	5 days	0.66	0.72	0.66	0.45	0.41	0.40	−0.25	−0.17	−0.22
	6 days	0.69	0.67	0.67	0.49	0.36	0.41	−0.26	−0.18	−0.23
	7 days	0.69	0.67	0.67	0.53	0.33	0.43	−0.23	−0.21	−0.22
										
*Random daytime casual urine*										
	1 day	0.61	0.64	0.61	0.32	0.43	0.35	−0.40	−0.14	−0.30
	2 days	0.71	0.56	0.65	0.40	0.35	0.36	−0.45	−0.13	−0.33
	3 days	0.65	0.66	0.64	0.24	0.27	0.23	−0.56	−0.29	−0.46
	4 days	0.76	0.76	0.75	0.23	0.33	0.24	−0.63	−0.32	−0.52
	5 days	0.79	0.77	0.77	0.24	0.32	0.25	−0.66	−0.34	−0.54
	6 days	0.83	0.82	0.81	0.32	0.43	0.33	−0.60	−0.26	−0.49
	7 days	0.87	0.85	0.85	0.34	0.43	0.35	−0.63	−0.29	−0.51
										
*Urine before bedtime*										
	1 day	0.39	0.69	0.49	0.25	0.23	0.25	−0.29	−0.35	−0.30
	2 days	0.40	0.81	0.54	0.16	0.33	0.24	−0.38	−0.36	−0.37
	3 days	0.42	0.84	0.57	0.17	0.44	0.31	−0.39	−0.28	−0.33
	4 days	0.56	0.87	0.67	0.23	0.45	0.34	−0.45	−0.29	−0.38
	5 days	0.59	0.85	0.68	0.28	0.44	0.37	−0.43	−0.27	−0.36
	6 days	0.58	0.82	0.66	0.29	0.37	0.34	−0.39	−0.33	−0.36
	7 days	0.59	0.79	0.66	0.26	0.33	0.31	−0.43	−0.35	−0.39
										
*Random casual urine* *(selected from each day)*										
	1 day	0.56	0.51	0.53	0.25	0.13	0.19	−0.37	−0.31	−0.35
	2 days	0.50	0.71	0.55	0.37	0.34	0.34	−0.18	−0.25	−0.20
	3 days	0.56	0.77	0.63	0.37	0.25	0.32	−0.29	−0.40	−0.33
	4 days	0.80	0.82	0.80	0.42	0.24	0.33	−0.50	−0.46	−0.49
	5 days	0.83	0.80	0.81	0.46	0.30	0.38	−0.49	−0.38	−0.45
	6 days	0.86	0.80	0.84	0.45	0.25	0.36	−0.53	−0.43	−0.49
	7 days	0.87	0.88	0.87	0.45	0.32	0.39	−0.53	−0.44	−0.50

Abbreviations: K, potassium; Na, sodium.

*P*<0.001 for all coefficient data collection.

a Means of all 7 days.
