# Supplementary material for: Four to seven random casual urine specimens are sufficient to estimate 24-h urinary sodium/potassium ratio in individuals with high blood pressure
Source: J Hum Hypertens. 2015 Aug 27;30(5):328–34. doi: 10.1038/jhh.2015.84 (PMC4827004; doi:10.1038/jhh.2015.84)
Supplement: Supplementary Information [file jhh201584x1.doc]

**Appendix Table 1.** Correlation coefficients of casual urine Na concentration with 7-day 24-hr Na/K ratio, Na excretion, and K excretion in 74 individuals with high blood pressure.

| Time of casual urine | Number of days to calculate mean | Correlation coefficients | | | | | | | | |
| --- | --- | --- | --- | --- | --- | --- | --- | --- | --- | --- |
|  |  | 24-hr Na/K ratio^a^ | | | 24-hr Na excretion^a^ (mmol/24 h) | | | 24-hr K excretion^a^ (mmol/24 h) | | |
|  |  | With medication (n=43) | Without medication (n=31) | Overall (n=74) | With medication (n=43) | Without medication (n=31) | Overall (n=74) | With medication (n=43) | Without medication (n=31) | Overall (n=74) |
| First morning urine | 1 day | 0.23 | 0.52 | 0.34 | 0.21 | 0.60 | 0.40 | -0.06 | 0.22 | 0.05 |
|  | 2 days | 0.28 | 0.57 | 0.39 | 0.30 | 0.64 | 0.48 | -0.02 | 0.20 | 0.08 |
|  | 3 days | 0.31 | 0.62 | 0.43 | 0.35 | 0.73 | 0.54 | 0.01 | 0.24 | 0.11 |
|  | 4 days | 0.29 | 0.61 | 0.42 | 0.34 | 0.72 | 0.53 | 0.02 | 0.24 | 0.12 |
|  | 5 days | 0.37 | 0.60 | 0.46 | 0.37 | 0.73 | 0.56 | -0.03 | 0.27 | 0.10 |
|  | 6 days | 0.32 | 0.57 | 0.41 | 0.37 | 0.73 | 0.54 | 0.02 | 0.29 | 0.13 |
|  | 7 days | 0.36 | 0.58 | 0.44 | 0.40 | 0.69 | 0.54 | 0.00 | 0.24 | 0.09 |
| Second morning urine | 1 day | 0.18 | 0.52 | 0.31 | 0.22 | 0.55 | 0.39 | -0.02 | 0.14 | 0.05 |
|  | 2 days | 0.19 | 0.53 | 0.32 | 0.27 | 0.51 | 0.40 | 0.00 | 0.09 | 0.04 |
|  | 3 days | 0.26 | 0.50 | 0.35 | 0.25 | 0.56 | 0.41 | -0.07 | 0.15 | 0.03 |
|  | 4 days | 0.27 | 0.51 | 0.36 | 0.23 | 0.56 | 0.39 | -0.07 | 0.13 | 0.02 |
|  | 5 days | 0.28 | 0.50 | 0.36 | 0.26 | 0.59 | 0.42 | -0.06 | 0.18 | 0.04 |
|  | 6 days | 0.28 | 0.43 | 0.34 | 0.29 | 0.61 | 0.44 | -0.03 | 0.26 | 0.09 |
|  | 7 days | 0.31 | 0.44 | 0.36 | 0.34 | 0.57 | 0.45 | -0.03 | 0.21 | 0.07 |
| Random daytime casual urine | 1 day | 0.53 | 0.52 | 0.52 | 0.48 | 0.47 | 0.48 | -0.16 | 0.05 | -0.07 |
|  | 2 days | 0.35 | 0.57 | 0.43 | 0.44 | 0.45 | 0.45 | 0.01 | -0.01 | 0.00 |
|  | 3 days | 0.38 | 0.59 | 0.46 | 0.42 | 0.52 | 0.46 | -0.07 | 0.03 | -0.03 |
|  | 4 days | 0.40 | 0.64 | 0.48 | 0.35 | 0.55 | 0.44 | -0.15 | 0.01 | -0.08 |
|  | 5 days | 0.39 | 0.62 | 0.47 | 0.40 | 0.58 | 0.47 | -0.08 | 0.06 | -0.02 |
|  | 6 days | 0.42 | 0.61 | 0.48 | 0.43 | 0.60 | 0.49 | -0.08 | 0.10 | -0.01 |
|  | 7 days | 0.44 | 0.61 | 0.49 | 0.43 | 0.60 | 0.50 | -0.11 | 0.11 | -0.02 |
| Urine before bedtime | 1 day | 0.17 | 0.49 | 0.30 | 0.15 | 0.43 | 0.31 | -0.12 | 0.05 | -0.04 |
|  | 2 days | 0.19 | 0.48 | 0.31 | 0.15 | 0.40 | 0.29 | -0.15 | 0.00 | -0.08 |
|  | 3 days | 0.21 | 0.59 | 0.36 | 0.20 | 0.49 | 0.36 | -0.13 | 0.01 | -0.06 |
|  | 4 days | 0.29 | 0.56 | 0.40 | 0.27 | 0.49 | 0.39 | -0.14 | 0.02 | -0.06 |
|  | 5 days | 0.33 | 0.58 | 0.43 | 0.34 | 0.56 | 0.46 | -0.12 | 0.09 | -0.02 |
|  | 6 days | 0.34 | 0.56 | 0.42 | 0.33 | 0.55 | 0.45 | -0.13 | 0.10 | -0.03 |
|  | 7 days | 0.36 | 0.53 | 0.43 | 0.38 | 0.49 | 0.45 | -0.10 | 0.06 | -0.03 |
| Random casual urine | 1 day | 0.32 | 0.11 | 0.23 | 0.41 | 0.44 | 0.42 | -0.04 | 0.37 | 0.15 |
| (Selected from each day) | 2 days | 0.31 | 0.34 | 0.32 | 0.43 | 0.55 | 0.49 | 0.02 | 0.29 | 0.14 |
|  | 3 days | 0.27 | 0.46 | 0.34 | 0.35 | 0.63 | 0.48 | -0.03 | 0.27 | 0.10 |
|  | 4 days | 0.38 | 0.47 | 0.41 | 0.41 | 0.58 | 0.49 | -0.08 | 0.18 | 0.04 |
|  | 5 days | 0.40 | 0.50 | 0.43 | 0.44 | 0.59 | 0.50 | -0.07 | 0.17 | 0.04 |
|  | 6 days | 0.41 | 0.52 | 0.44 | 0.46 | 0.59 | 0.52 | -0.04 | 0.15 | 0.04 |
|  | 7 days | 0.45 | 0.49 | 0.45 | 0.52 | 0.60 | 0.52 | 0.03 | 0.19 | 0.04 |

^a^ Means of all 7 days

Na, sodium; K, potassium.

P<0.001 for all coefficient

**Appendix Table 2.** Correlation coefficients of casual urine K concentration with 7-day 24-hr Na/K ratio, Na excretion, and K excretion in 74 individuals with high blood pressure

| Time of casual urine | Number of days to calculate mean | Correlation coefficients | | | | | | | | |
| --- | --- | --- | --- | --- | --- | --- | --- | --- | --- | --- |
|  |  | 24-hr Na/K ratio^a^ | | | 24-hr Na excretion^a^ (mmol/24 h) | | | 24-hr K excretion^a^ (mmol/24 h) | | |
|  |  | With medication (n=43) | Without medication (n=31) | Overall (n=74) | With medication (n=43) | Without medication (n=31) | Overall (n=74) | With medication (n=43) | Without medication (n=31) | Overall (n=74) |
| First morning urine | 1 day | -0.09 | -0.23 | -0.14 | -0.02 | 0.13 | 0.04 | 0.06 | 0.37 | 0.18 |
|  | 2 days | -0.18 | -0.21 | -0.19 | -0.06 | 0.16 | 0.02 | 0.14 | 0.39 | 0.24 |
|  | 3 days | -0.31 | -0.24 | -0.29 | -0.15 | 0.24 | -0.01 | 0.20 | 0.51 | 0.30 |
|  | 4 days | -0.33 | -0.23 | -0.31 | -0.16 | 0.28 | -0.02 | 0.20 | 0.54 | 0.31 |
|  | 5 days | -0.27 | -0.24 | -0.27 | -0.12 | 0.27 | 0.00 | 0.18 | 0.51 | 0.28 |
|  | 6 days | -0.29 | -0.27 | -0.29 | -0.12 | 0.26 | -0.01 | 0.19 | 0.53 | 0.29 |
|  | 7 days | -0.26 | -0.30 | -0.28 | -0.11 | 0.22 | -0.02 | 0.16 | 0.51 | 0.26 |
| Second morning urine | 1 day | -0.24 | -0.03 | -0.17 | -0.25 | 0.11 | -0.10 | 0.00 | 0.15 | 0.05 |
|  | 2 days | -0.20 | -0.02 | -0.13 | -0.13 | 0.09 | -0.04 | 0.09 | 0.10 | 0.09 |
|  | 3 days | -0.32 | -0.09 | -0.23 | -0.19 | 0.08 | -0.06 | 0.18 | 0.15 | 0.17 |
|  | 4 days | -0.36 | -0.15 | -0.28 | -0.22 | 0.04 | -0.10 | 0.20 | 0.14 | 0.18 |
|  | 5 days | -0.40 | -0.20 | -0.32 | -0.26 | 0.03 | -0.13 | 0.21 | 0.18 | 0.19 |
|  | 6 days | -0.46 | -0.20 | -0.36 | -0.25 | 0.08 | -0.10 | 0.27 | 0.24 | 0.26 |
|  | 7 days | -0.44 | -0.21 | -0.34 | -0.27 | 0.06 | -0.11 | 0.25 | 0.22 | 0.24 |
| Random daytime casual urine | 1 day | -0.38 | -0.18 | -0.31 | -0.22 | -0.08 | -0.16 | 0.22 | 0.07 | 0.16 |
|  | 2 days | -0.44 | -0.06 | -0.30 | -0.14 | -0.02 | -0.08 | 0.39 | 0.00 | 0.24 |
|  | 3 days | -0.39 | -0.13 | -0.29 | -0.06 | 0.04 | -0.01 | 0.40 | 0.14 | 0.29 |
|  | 4 days | -0.46 | -0.21 | -0.38 | -0.11 | 0.00 | -0.06 | 0.40 | 0.16 | 0.32 |
|  | 5 days | -0.49 | -0.22 | -0.40 | -0.07 | 0.04 | -0.03 | 0.49 | 0.22 | 0.39 |
|  | 6 days | -0.49 | -0.28 | -0.43 | -0.11 | -0.02 | -0.08 | 0.45 | 0.19 | 0.36 |
|  | 7 days | -0.52 | -0.29 | -0.45 | -0.13 | 0.02 | -0.08 | 0.45 | 0.27 | 0.38 |
| Urine before bedtime | 1 day | -0.25 | -0.01 | -0.18 | -0.09 | 0.24 | 0.03 | 0.28 | 0.29 | 0.28 |
|  | 2 days | -0.28 | -0.24 | -0.27 | -0.07 | 0.05 | -0.03 | 0.30 | 0.29 | 0.30 |
|  | 3 days | -0.27 | -0.28 | -0.27 | -0.04 | 0.04 | -0.02 | 0.30 | 0.31 | 0.30 |
|  | 4 days | -0.29 | -0.32 | -0.30 | -0.03 | 0.02 | -0.03 | 0.31 | 0.32 | 0.31 |
|  | 5 days | -0.27 | -0.30 | -0.28 | -0.03 | 0.08 | 0.00 | 0.29 | 0.40 | 0.32 |
|  | 6 days | -0.28 | -0.33 | -0.30 | -0.04 | 0.13 | 0.01 | 0.30 | 0.47 | 0.34 |
|  | 7 days | -0.30 | -0.31 | -0.30 | -0.01 | 0.10 | 0.01 | 0.34 | 0.43 | 0.36 |
| Random casual urine | 1 day | -0.25 | -0.25 | -0.25 | 0.03 | 0.14 | 0.09 | 0.29 | 0.39 | 0.33 |
| (Selected from each day) | 2 days | -0.32 | -0.39 | -0.34 | -0.07 | -0.03 | -0.04 | 0.28 | 0.31 | 0.29 |
|  | 3 days | -0.31 | -0.39 | -0.33 | -0.06 | 0.08 | -0.01 | 0.32 | 0.42 | 0.36 |
|  | 4 days | -0.49 | -0.37 | -0.45 | -0.11 | 0.13 | -0.02 | 0.51 | 0.45 | 0.49 |
|  | 5 days | -0.53 | -0.36 | -0.48 | -0.13 | 0.10 | -0.05 | 0.53 | 0.40 | 0.48 |
|  | 6 days | -0.56 | -0.38 | -0.51 | -0.13 | 0.13 | -0.05 | 0.57 | 0.46 | 0.52 |
|  | 7 days | -0.58 | -0.43 | -0.53 | -0.13 | 0.11 | -0.05 | 0.59 | 0.49 | 0.54 |

^a^ Means of all 7 days

Na, sodium; K, potassium.

P<0.001 for all coefficient data collection

**Appendix Table 3.** Correlation coefficients of 1 to 7-day 24-hr Na/K ratio, Na excretion, and K excretion with 7-day 24-hr Na/K ratio, Na excretion, and K excretion in 74 individuals with high blood pressure

| Time of casual urine | Number of days to calculate mean | Correlation coefficients | | | | | | | | |
| --- | --- | --- | --- | --- | --- | --- | --- | --- | --- | --- |
|  |  | 24-hr Na/K ratio^a^ | | | 24-hr Na excretion^a^ (mmol/24 h) | | | 24-hr K excretion^a^ (mmol/24 h) | | |
|  |  | With medication (n=43) | Without medication (n=31) | Overall (n=74) | With medication (n=43) | Without medication (n=31) | Overall (n=74) | With medication (n=43) | Without medication (n=31) | Overall (n=74) |
| 24-hr Na/K ratio | 1 day | 0.74 | 0.79 | 0.75 | 0.61 | 0.49 | 0.56 | -0.28 | -0.16 | -0.23 |
|  | 2 days | 0.84 | 0.88 | 0.86 | 0.64 | 0.55 | 0.60 | -0.36 | -0.18 | -0.30 |
|  | 3 days | 0.87 | 0.93 | 0.89 | 0.59 | 0.53 | 0.57 | -0.45 | -0.24 | -0.37 |
|  | 4 days | 0.94 | 0.94 | 0.94 | 0.58 | 0.50 | 0.54 | -0.51 | -0.28 | -0.43 |
|  | 5 days | 0.97 | 0.96 | 0.97 | 0.57 | 0.50 | 0.54 | -0.54 | -0.30 | -0.45 |
|  | 6 days | 0.99 | 0.98 | 0.99 | 0.56 | 0.53 | 0.55 | -0.56 | -0.29 | -0.46 |
|  | 7 days | 1.00 | 1.00 | 1.00 | 0.56 | 0.53 | 0.54 | -0.58 | -0.31 | -0.48 |
| 24-hr Na excretion | 1 day | 0.41 | 0.42 | 0.42 | 0.80 | 0.86 | 0.83 | 0.27 | 0.58 | 0.39 |
|  | 2 days | 0.41 | 0.54 | 0.46 | 0.87 | 0.92 | 0.89 | 0.32 | 0.54 | 0.40 |
|  | 3 days | 0.41 | 0.53 | 0.46 | 0.90 | 0.95 | 0.93 | 0.37 | 0.58 | 0.45 |
|  | 4 days | 0.52 | 0.58 | 0.54 | 0.95 | 0.96 | 0.96 | 0.30 | 0.54 | 0.40 |
|  | 5 days | 0.54 | 0.56 | 0.55 | 0.97 | 0.98 | 0.98 | 0.31 | 0.58 | 0.42 |
|  | 6 days | 0.58 | 0.54 | 0.56 | 0.99 | 0.99 | 0.99 | 0.30 | 0.61 | 0.43 |
|  | 7 days | 0.57 | 0.53 | 0.55 | 1.00 | 1.00 | 1.00 | 0.31 | 0.63 | 0.44 |
| 24-hr K excretion | 1 day | -0.46 | -0.18 | -0.35 | 0.26 | 0.56 | 0.40 | 0.80 | 0.80 | 0.80 |
|  | 2 days | -0.54 | -0.23 | -0.43 | 0.25 | 0.58 | 0.39 | 0.89 | 0.87 | 0.88 |
|  | 3 days | -0.53 | -0.30 | -0.44 | 0.30 | 0.58 | 0.42 | 0.95 | 0.94 | 0.94 |
|  | 4 days | -0.54 | -0.27 | -0.44 | 0.31 | 0.62 | 0.44 | 0.97 | 0.95 | 0.96 |
|  | 5 days | -0.54 | -0.28 | -0.44 | 0.33 | 0.64 | 0.47 | 0.99 | 0.98 | 0.98 |
|  | 6 days | -0.57 | -0.28 | -0.46 | 0.30 | 0.65 | 0.46 | 0.99 | 0.99 | 0.99 |
|  | 7 days | -0.57 | -0.31 | -0.47 | 0.31 | 0.63 | 0.44 | 1.00 | 1.00 | 1.00 |

^a^ Means of all 7 days

Na, sodium; K, potassium.

P<0.001 for all coefficient data collection
